# The Olfactory Trail of Neurodegenerative Diseases

**DOI:** 10.3390/cells13070615

**Published:** 2024-04-02

**Authors:** Rafael Franco, Claudia Garrigós, Jaume Lillo

**Affiliations:** 1 Department of Biochemistry and Molecular Biomedicine, Faculty of Biology, University of Barcelona, 08028 Barcelona, Spain; jaumelillo@ub.edu; 2 CiberNed, Network Center for Neurodegenerative Diseases, National Spanish Health Institute Carlos III, 28029 Madrid, Spain; 3 School of Chemistry, University of Barcelona, 08028 Barcelona, Spain

**Keywords:** olfactory receptors, ectopic expression, Parkinson’s disease, Alzheimer’s disease, neurons, microglia

## Abstract

Alterations in olfactory functions are proposed as possible early biomarkers of neurodegenerative diseases. Parkinson’s and Alzheimer’s diseases manifest olfactory dysfunction as a symptom, which is worth mentioning. The alterations do not occur in all patients, but they can serve to rule out neurodegenerative pathologies that are not associated with small deficits. Several prevalent neurodegenerative conditions, including impaired smell, arise in the early stages of Parkinson’s and Alzheimer’s diseases, presenting an attractive prospect as a snitch for early diagnosis. This review covers the current knowledge on the link between olfactory deficits and Parkinson’s and Alzheimer’s diseases. The review also covers the emergence of olfactory receptors as actors in the pathophysiology of these diseases. Olfactory receptors are not exclusively expressed in olfactory sensory neurons. Olfactory receptors are widespread in the human body; they are expressed, among others, in the testicles, lungs, intestines, kidneys, skin, heart, and blood cells. Although information on these ectopically expressed olfactory receptors is limited, they appear to be involved in cell recognition, migration, proliferation, wound healing, apoptosis, and exocytosis. Regarding expression in non-chemosensory regions of the central nervous system (CNS), future research should address the role, in both the glia and neurons, of olfactory receptors. Here, we review the limited but relevant information on the altered expression of olfactory receptor genes in Parkinson’s and Alzheimer’s diseases. By unraveling how olfactory receptor activation is involved in neurodegeneration and identifying links between olfactory structures and neuronal death, valuable information could be gained for early diagnosis and intervention strategies in neurodegenerative diseases.

## 1. Introduction

Loss of smell is often associated with neurodegenerative diseases. Olfactory deficits do not occur in all patients but can occur in patients with Alzheimer’s disease, Parkinson’s disease, Huntington’s disease, or schizophrenia. When the alteration of smell appears, it usually does so before the most common symptoms of the disease, that is, in the prodromal phase; it is unknown whether it is a consequence of the disease or on the contrary, a dysfunctional olfactory system promoting neuronal death in different areas of the brain through circuit alterations. Another aspect of the olfactory pathway to neurodegenerative diseases is the finding of olfactory receptors in non-sensory areas of the brain. Future research should shed light on what these ectopically expressed receptors “smell” there, and on the possible role of olfactory receptors in the pathophysiology of neurological and neuropsychiatric diseases.

This review considers both sides of the relationship between olfaction and the pathophysiology of Parkinson’s and Alzheimer’s diseases. One relates to the causes of the alteration in smell perception in the early stages of neurodegenerative diseases, and considers what the consequences could be in terms of symptoms and speed of disease progression. Another considers information related to the expression of olfactory receptors in non-chemosensory areas of the central nervous system (CNS). The two most relevant questions that should be addressed in future research are as follows: (1) Do ectopic olfactory receptors expressed in the brain contribute to the pathogenesis and/or progression of Parkinson’s and Alzheimer’s diseases? and (2) can olfactory receptors be therapeutic targets for these diseases? Particularly relevant is to address the possible neuroprotective effect, if any, of targeting ectopic CNS olfactory receptors.

## 2. The Olfactory System and the Pathophysiology of Neurodegenerative Diseases

Several findings underscore the integral role of olfactory structures in neurodegenerative processes, unraveling a narrative that extends beyond mere olfactory function [1].

An intriguing facet is the olfactory dysfunction that emerges in some patients with neurodegenerative diseases [2]. Is this a consequence of interactions between the olfactory bulb and the areas that degenerate in, among others, Parkinson’s disease and dementia?

**Figure 1:**
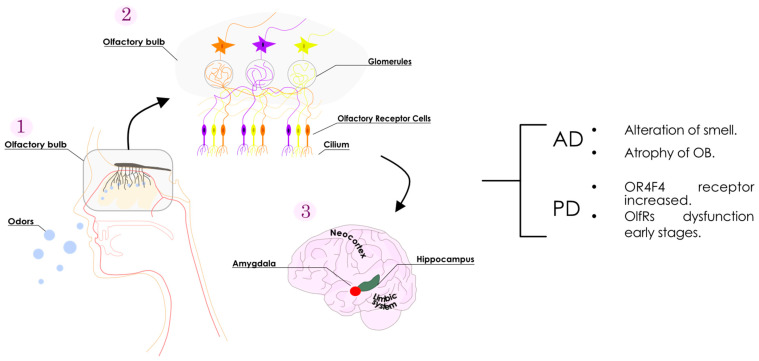
Schematic representation of the olfactory system. Odorants arrive at the nasal mucosa (1), and progress towards the olfactory receptors in the cilium (2). Neurons containing olfactory receptors transmit the information from the cilium to the olfactory bulb via the glomeruli (3). The olfactory bulb receives and decodes the information. The processed data are then directly dispatched to the limbic system (involving the amygdala and hippocampus) and the neocortex for further information processing. Olfactory receptor-related alterations reported for the main neurodegenerative diseases are as summarized (right).

Within the complex olfactory processing network (Figure 1), the olfactory bulb is the primary place for deciphering olfactory information in the brain. Emerging from the olfactory epithelium, the axons of olfactory receptor neurons embark toward the brain and finally penetrate the olfactory bulb [3]. In the olfactory bulb, the axons of so-called “receptor cells” form synapses with the dendrites of other cells, forming what is believed to be the initial link for the processing of olfactory signals [4]. Subsequently, the olfactory tract is formed as these axons emanate from the olfactory bulb, unfolding in collateral branches that extend towards the olfactory cortex where the complex processing of olfactory information takes place [1,5,6].

### 2.1. Olfaction, Olfactory Receptors, and Parkinson’s Disease

Parkinson’s disease arises due to the progressive death of dopaminergic neurons in the substantia nigra. Most cases are sporadic, but some are hereditary and result from mutations in, among others, genes of the alpha-synuclein (SNCA), leucine-rich repeat kinase 2 (LRRK2), parkin (PARK2) or PTEN-induced kinase 1 (PINK1) [7,8,9].

In the late 1960s, a significant shift occurred in the landscape of Parkinson’s disease treatment, years after the identification of dopamine as the deficient neurotransmitter. The first clinical trials involving levodopa (L-DOPA) marked a turning point, by affording efficacious therapy for addressing motor symptoms [10,11,12]. This success, along with efforts to identify preclinical symptoms for early disease recognition and intervention, has had far-reaching consequences; management of the disease is now very efficient, even though there has been little progress in preventing disease progression [13]. There is interest in the early detection of Parkinson’s disease by providing tools, biomarkers included, that appear sooner than motor manifestations. Early indicators that lead to suspicion of the death of nigral dopaminergic neurons include the following: (1) olfactory impairments, (2) disrupted sleep patterns, (3) diverse neuro-vegetative symptoms and (4) depression. Given the progressive nature of the disease, alterations involving motor control circuits can spread to other regions of the brain, leading to specific forms of cognitive impairment, mood irregularities, and, in some cases, dementia years after the appearance of motor symptoms [13,14,15].

Olfactory dysfunction manifests even in the premotor stages of the neurodegenerative process [16]. Olfactory manifestations in patients may differ depending on whether Parkinson’s disease is hereditary or sporadic (see [16] for an exhaustive review). In sporadic cases, olfactory tests may be more helpful in diagnosing Parkinson’s disease than motor scores [17,18]. These tests have been used to differentiate the disease from others that do not cause olfactory deficits, such as essential tremor, multiple system atrophy, or progressive supranuclear palsy [17,19,20,21,22,23].

Anosmia is not always present in patients with sporadic/idiopathic Parkinson’s disease. In one of the first studies focusing on this topic, 38% of 81 patients suffered from anosmia [24]. The correct perception of smell was frequently affected. Furthermore, the alteration was bilateral and transversal, that is, the deficit was not related to a specific type of odor but was reported for all odorants used in the tests [17,18,25,26,27,28,29,30].

In the monogenic hereditary disease, olfactory alterations have been found, but “randomly”, that is, not all those affected by the same mutation present olfactory deficits (see [16] for more details). As an illustrative example, two out of the seven Greek patients with the G^209^A mutation in the SNCA gene had an abnormal olfactory function, even though in the other five, it was normal [31].

There is still debate on whether olfactory dysfunction is a cause or a consequence of the disease and there are few hypotheses on the potential role of the olfactory system in aggravating neurodegenerative disease progression. One of the hypotheses derives from the increased risk of developing Parkinson’s disease in individuals with a mutation in the CYP2D6-debrisoquine hydroxylase gene [32,33]. This type of enzymatic activity is found in the olfactory mucosa and therefore reduced enzyme activity, resulting in reduced xenobiotic inactivation that can lead to nasal penetration of toxic molecules into the brain [34,35,36,37]. It is intriguing why Lewy bodies can appear early in the cells that innervate the stomach and olfactory bulb. Consequently, it is suggested that a neurotrophic virus can go from these places to the brain, that is, two routes are suggested: from the gastric choroid plexus to the brain, and from the nose to the temporal lobe by anterograde transport. This is known as the dual-hit hypothesis; the proponents wrote: “*the most parsimonious explanation for the initial events of sporadic Parkinson’s disease is pathogenic access to the brain through the stomach and nose—hence the term ‘dual-hit’*” [38]. However attractive, all hypotheses are difficult to test, and, to our knowledge, none has reached consensus.

### 2.2. Olfaction, Olfactory Receptors, and Alzheimer’s Disease

The criteria for making a diagnosis of Alzheimer’s disease have undergone alterations over time. The original definition, pioneered by Alois Alzheimer, described it as a form of presenile cognitive impairment, involving symptoms before age 65. This condition was characterized by the accumulation of extracellular structures called senile plaques and of intracellular neurofibrillary tangles. Senile plaques, consisting of an amyloid core with neurofibrillary tangles composed of hyperphosphorylated and aggregated tau, remain hallmark features of Alzheimer’s disease, regardless of the age at which clinical symptoms appear.

Alzheimer’s disease is now clinically classified as prodromic or preclinical (without significant symptoms), mild cognitive impairment of the Alzheimer’s disease type, and dementia of the Alzheimer’s type. Robust efforts are currently being made to identify early biomarkers to make the diagnosis as early as possible [39,40,41].

The identification of substantial axonal degeneration in the olfactory tract of individuals with Alzheimer’s disease is noteworthy; morphological and functional alterations found in the human olfactory nerve (obtained post-mortem) suggest that imbalances could start in the olfactory system and propagate towards the hippocampus and the entorhinal cortex [2]. Additionally, there is atrophy in the olfactory bulb in patients with Alzheimer’s disease, as determined by high-resolution magnetic resonance imaging. Decreased volume in this area correlates with reduced density in temporal lobe gray matter [42,43].

Current knowledge reveals that a certain percentage of patients have what is known as early-onset Alzheimer’s disease (EOAD). EOAD is hereditary and mainly associated with mutations in three genes, those coding for the (1) amyloid precursor protein (APP), (2) presenilin 1 (PSEN1), and (iii) presenilin 2 (PSEN2). Late-onset Alzheimer’s disease (LOAD) is considered sporadic, and age is the main risk factor. The risk of LOAD may increase due to environmental factors, behavioral factors, and genetic factors. Studies have consistently shown that possession of the ε4 allele is a risk factor for LOAD. The APOE gene has three main alleles, ε2, ε3, and ε4. Individuals with one or two copies of the ε4 allele are at an increased risk of developing Alzheimer’s disease compared to those without the ε4 allele. In contrast, the ε2 allele may have a protective effect [44]. Like other neurodegenerative conditions featuring abnormal protein aggregates, Alzheimer’s disease develops as a prolonged progression, in which cognitive decline and dementia only become evident after substantial neuronal death and brain shrinkage have occurred [45].

Along with the well-known manifestations, that is, cognitive impairment and behavioral alterations, a significant number of people diagnosed with Alzheimer’s disease present an alteration of smell [46]. However, the origin of this olfactory dysfunction is unknown. Neurological examinations bolster the notion that early events in Alzheimer’s disease pathophysiology involve the aggregation of hyperphosphorylated tau and Aβ in the olfactory bulb, leading to a failure to maintain homeostasis [47]. Nonetheless, the mechanisms through which progressive amyloidogenic pathology influences the functionality of the olfactory bulb remain unclear.

A deeper understanding of the molecular processes affected by the progressive accumulation of amyloid pathology could reveal new targets in the olfactory pathways for earlier diagnostics and therapeutic interventions. In Tg2576 transgenic mice, alterations in the olfactory bulb arise at stages before plaque formation, and the functional interactome of the amyloid precursor protein undergoes progressive modulation at the olfactory level [47]. Furthermore, different olfactory signaling pathways (Akt, p38, MAPK, SEK1 and SAPK) undergo time-dependent modulation compared to WT animals. However, SEK1 and PKA pathways are differentially activated during the grading of human Alzheimer’s disease at the level of the olfactory bulb [47]. These revelations at the early pre-plaque stage offer mechanistic insights into the olfactory mechanisms associated with the progression of cognitive deficits previously documented in mice that were models of Alzheimer’s disease [48].

Based on meta-analysis studies, Shady and colleagues (2012) reported that both Alzheimer’s disease and Parkinson’s disease affect odor identification more than odor detection itself [48]. Although neuronal death occurs in different locations in these two diseases, another meta-analysis concluded that the mechanism underlying olfactory dysfunction may be shared in both Alzheimer’s and Parkinson’s diseases [49].

## 3. Olfactory Receptors

The significance of olfactory receptors in the pathophysiology of neurological diseases is an emerging area of research that underscores a link between the olfactory system and various neurological conditions. Olfactory receptors, traditionally associated with the sense of smell, are now recognized for their presence and functional roles beyond the olfactory epithelium, extending into the central nervous system. Olfactory receptors have been investigated in the context of neurodegenerative processes, including altered proteotranscriptomic patterns in functional interactomes. Understanding how olfactory receptors are modulated during disease progression provides potential avenues for early diagnosis and therapeutic interventions.

**Figure 2:**
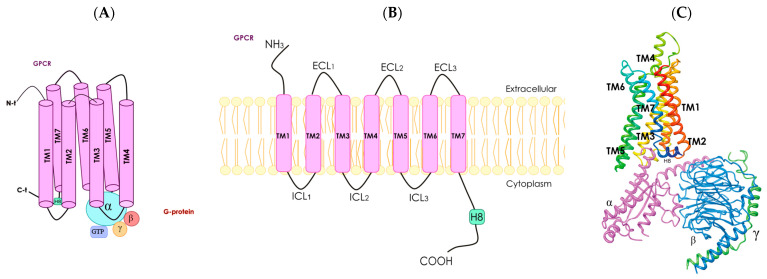
Schematic representation of a G protein-coupled receptor. Panel (**A**), diagram showing the seven transmembrane (TM) α-helices, α helix 8 (H8), N-terminal (N-t) extracellular-facing domain and C-terminal (C-t) cytoplasmic-facing domain. G protein-coupled receptors are associated with heterotrimeric G protein (with α, ß and γ subunits). Panel (**B**), two-dimensional representation of a G protein-coupled receptor; the seven TM α-helices are connected by intracellular (ICL1–ICL3) and extracellular (ECL1–ECL3) loops. Panel (**C**), three-dimensional structure of a receptor coupled with a G protein with α (fuchsia), ß (blue) and γ (green) subunits (from PDB Protein Database, structure identification number: 6D9H).

Mammalian olfactory receptors (olfactory receptors) are considered to belong to the family of G protein-coupled receptors (Figure 2) [50]. However, pheromone perception in insects is mediated by odor-gated ion channels [51]. Bioinformatics analysis provided evidence that insect olfactory sensory cells contained receptors similar to mammalian ionotropic glutamate receptors. Later research demonstrated that these receptors, which lacked glutamate-interacting amino acid residues, were involved in chemosensory actions [51,52]. Although there is no ionotropic receptor in mammals that is directly involved in the sense of smell, it seems plausible that ionotropic receptors or channels in the olfactory epithelia may amplify the signal provided in mammals by the olfactory receptors [53]. His review focuses on mammalian olfactory receptors that have the well-known G protein-coupled receptor structure (Figure 2).

G protein-coupled receptors constitute the most populated family of the human proteome. They share structural features and the possibility of interaction with heteromeric G proteins, which are constituted of α, ß and γ subunits (Figure 2). A common structural feature is the set of seven alpha helices that span the cell membrane. Although G protein-coupled receptors can be found intracellularly, their function is mainly exerted in the plasma membrane. In fact, G protein-coupled receptors sense the environment for the cells to respond appropriately. Each of the existing hundreds of G protein-coupled receptors respond to a given molecule. Structures of the activators (agonists) of G protein-coupled receptors can be quite diverse, ranging from amino acids to lipids. Furthermore, G protein-coupled receptors can be activated by photons. Upon activation, there are signal transduction mechanisms mainly mediated by heteromeric G proteins; there are several genes that code for G proteins. Depending on the α subunit, G proteins may associate with, for instance, the cAMP-protein kinase A (PKA) or the Ca^2+^-protein kinase C (PKC) signaling pathways. In summary, activation of G protein-coupled receptors leads to the activation of diverse heteromeric G proteins that are linked to different signal transduction mechanisms thus leading to ad hoc cell responses (Figure 2).

The following are the four main families of heterotrimeric G proteins: G_s_, G_i/o_, G_q_ and G_12/13_. G_s_ proteins contain α_s_ subunits and their engagement in signaling leads to the activation of the adenylate cyclase, the enzyme that converts ATP into cyclic adenosine monophosphate (cAMP). In contrast, G_i_ proteins contain α_i_ subunits that, upon engagement, inhibit the adenylate cyclase. G_q_ proteins contain α_q_ subunits that, when activated, increase the catalytic activity of phospholipase C, consequently increasing inositol 1,4,5-triphosphate (IP_3_) levels and releasing Ca^2+^ from the endoplasmic reticulum to the cytoplasm. The role of G_12/13_ proteins in signal transduction seems to be mediated by Rho-type proteins (see [54] for review).

## 4. Discovery of Olfactory Receptors as G Protein-Coupled Receptors

**Figure 3:**
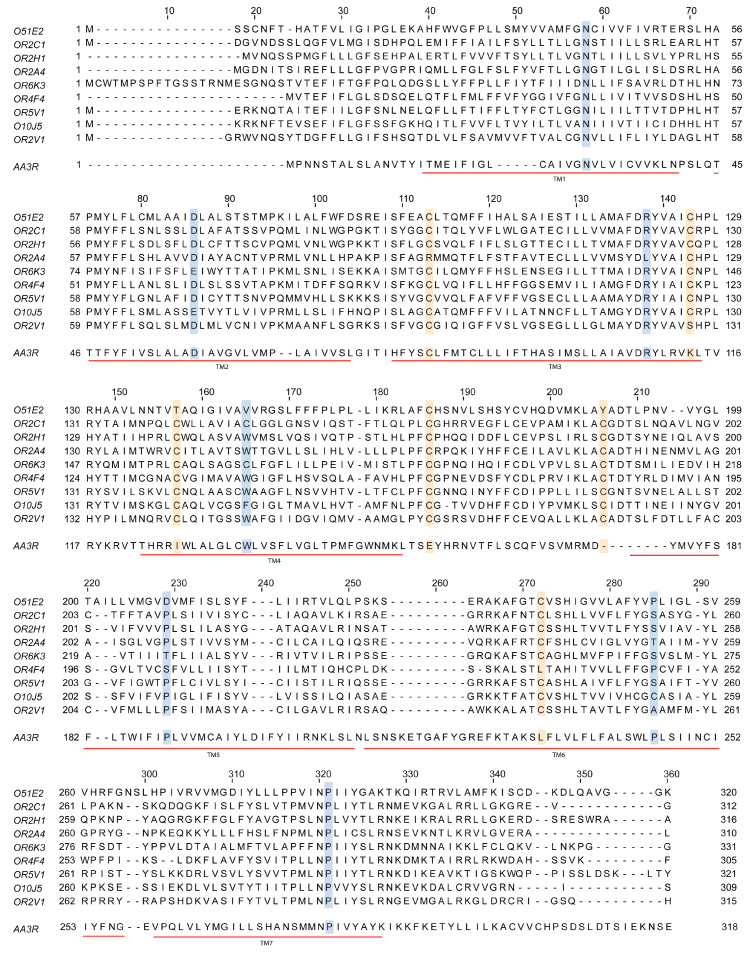
Sequence comparison of some olfactory receptors and the adenosine A_3_ receptor, which is a class A G protein-coupled receptor expressed in virtually all organs and tissues. Sequences of the olfactory receptors mentioned in this review are aligned with that of the human adenosine A_3_ receptor. Notice the presence of seven transmembrane α-helices in all cases (TM1 to TM7; underlined in red) and the highly conserved residues; conserved cysteines are in yellow and other conserved residues are in blue (some substitutions can be found for some of the olfactory receptors in these highly conserved positions).

The discovery of olfactory receptors, also known as odorant receptors, was achieved by looking for proteins differentially expressed in the olfactory system. With some exceptions, cloning of those proteins was not previously reported in other location(s) of the mammalian body, and thus led to the assumption that they were necessary for olfaction. Remarkably, the primary structure deduced from the gene sequences revealed homology with G protein-coupled receptors (Figure 3). The seven TM α-helices are always present and similar in length; in addition, there are highly conserved residues in these TM domains (Figure 3). The recent first-ever solved tertiary structure of an olfactory receptor confirmed the seven transmembrane helices domain and the likely coupling to heteromeric G proteins [55].

Classically, it has been assumed that olfactory receptors couple to heterotrimeric proteins known as G_olf_ that were detected in the olfactory system. The effect of G_olf_ proteins is similar to that caused by G_s_ proteins; their activation being associated with greater adenylate cyclase activity. In fact, the degree of homology between α subunits of G_olf_ and of G_s_ is extremely high, raising questions about whether G_olf_ and G_s_ are members of a different family. In addition, reports are suggesting that olfactory receptors may couple to G proteins other than G_s_ or G_olf_.

Until recently, interest in knowing more about olfactory receptors was purely academic. Olfaction is an important sense and, therefore, olfactory receptors have been instrumental in evolution because they help to, among other things, find food, avoid contaminated water or food, and flee from predators. However, unlike other G protein-coupled receptor subfamilies, olfactory receptors were not considered as a therapeutic target of any disease. When trying to learn more about olfactory receptors and their signaling mechanisms, problems arise, mainly derived from the lack of adequate pharmacological tools. The scenario is progressively changing and interest in olfactory receptors is now high, derived mainly from the identification of ectopic expression of the olfactory receptors, that is, their presence in cells outside the olfactory system.

### 4.1. Odorants as Activators of Olfactory Receptors

Merriam–Webster defines odorant as “*an odorous substance*”. The definition implies a molecule in the air that arrives at the human nose and is sensed as odorous. The molecule must activate some component in the olfactory system; therefore, it is hypothesized that olfactory receptors are those that detect odors and transmit information to the central nervous system (CNS). The CNS must perform the following: (1) notice that the odor is in the environment, and (2) establish mechanisms to remember the odor when it returns. Assumptions come from logical thinking but cannot be demonstrated scientifically due to the lack of appropriate tools. First, odorants are volatile, and it is difficult to demonstrate that a given odorant activates a given olfactory receptor. Second, at present, no rationale supports the synthesis of new molecules that selectively target an olfactory receptor. This scenario has just changed with the resolution of the structure of an olfactory receptor [55] because it is now possible for such a receptor, OR51E2, to make predictions by virtual screening.

It is important to consider that olfactory receptors are defined as being able to sense external molecules. This is in contrast with most members of the G protein-coupled receptor family that respond to endogenous molecules such as dopamine, adrenalin, serotonin, and chemokines. For comparison, taste receptors are, like olfactory receptors, designed to sense exogenous molecules. One wonders why a few taste receptors can perform the function of sensing and mediating the recognition of countless flavors in consumed foods, while hundreds of olfactory receptors are needed to detect and mediate the recognition of odors. Are all olfactory receptors sensing odors?

### 4.2. Reasons Why Olfactory Receptors Are in a G Protein-Coupled Receptor Limbo

Years ago, olfactory receptors had their own category within the G protein-coupled receptor family pharmacological database. A visit to the International Union of Pharmacology and British Pharmacological Society (IUPHAR/BPS) webpage (https://www.guidetopharmacology.org/; accessed on 14 January 2024), around 800 G protein-coupled receptors have been identified in humans: 33 are for taste and around 400 for olfaction. Although it is known that they are structurally like rhodopsin and, therefore, are of class A, they are not listed within class A. IUPHAR/BPS presents the following categories for G protein-coupled receptors: Class A, Class B, Class C, opsin receptors, taste 1 and taste 2. Olfactory receptors are not included in any of those categories; neither are they included in the “other 7TM proteins” category. The reason is found in this specific IUPHAR/BPS page (https://www.guidetopharmacology.org/GRAC/FamilyDisplayForward?familyId=694&familyType=GPCR; accessed on 14 January 2024): “*Odorant receptors are also seven-transmembrane spanning G protein-coupled receptors, responsible for the detection of odorant, generally volatile compounds associated with olfaction. These are not currently included as they are not yet associated with extensive pharmacological data but are curated in the following databases: The gene list of odorant receptors at HGNC, and curated by HORDE and ORDB*”.

### 4.3. Olfactory Receptor Nomenclature Is Diverse among Species

There is a well-established nomenclature for human olfactory receptors based on sequence similarity. They are classified into 18 families. Genes are marked with the letters “OR,” which stands for Olfactory Receptor, followed by a number representing the family, a letter for the subfamily, and a final number indicating the gene within the subfamily. For example, OR51E2, is member 2 of subfamily E within family 51 [56]. A gene group hierarchy map of the 18 families can be visualized in the HUGO gene nomenclature committee (HGNC) database (https://www.genenames.org/data/genegroup/#!/group/141; accessed on 23 January 2024). Pseudogenes are indicated by placing a `P’ at the end of the name, for instance: OR6B3P.

In other species, the nomenclature system varies. For instance, for mice, the ORDB database (https://ordb.biotech.ttu.edu/ORDB/; accessed on 23 January 2024) uses the prefix “MOR”, followed by a number corresponding to the family, a hyphen (-) and a number. A “P” at the end of the name indicates a pseudogene (e.g., MOR103-13P).

Whereas the names of genes and protein products are similar in humans and other species, the protein nomenclature of olfactory receptors is very diverse. In mice, the name of the receptor begins with ‘Olfr-’ followed by a number. Notice that the human ortholog of the Olfr78 receptor is OR51E2 (https://www.informatics.jax.org/marker/MGI:2157548; accessed on 23 January 2024). Continuing with the same human receptor, OR51E2, the ortholog in *Mus musculus* is known as Or51e2 or as Olfr78, the ortholog in *Xenopus tropicalis* is known as or51ao2, and the ortholog *Danio rerio* is known as or55e1 (https://www.alliancegenome.org/search?q=Or51e2, accessed on 30 March 2024). Matters become more convoluted because olfactory receptors previously identified in non-olfactory organs received names that were used for several years. For instance, OR51E2, was discovered in prostate cancer cells and named ‘prostate-specific G-protein-coupled receptor (PSGR)’ [57]. In summary, when searching for information about a given olfactory receptor, one may wish to search using all the synonyms in the databases.

## 5. Ectopic Expression of Olfactory Receptors

One of the classic desires in the biological sciences is that a given molecule, be it a protein, a metabolite, a hormone, or a neurotransmitter, is only expressed/produced in a given system or a given cell type. Reality indicates that these cases are exceptions and not the rule. To date, examples of exceptions are insulin production only in the pancreas or prostate-specific antigen, a marker of prostate cancer.

G protein-coupled receptors are expressed in different cell types in a variety of systems/organs. An example that we know first-hand is the CB_2_ cannabinoid receptor, which is expressed in peripheral cells and in the CNS, where it was supposedly expressed in the glia but not in neurons. Since we doubted whether the neurons were not expressing the CB_2_-type cannabinoid receptor, we performed experiments that confirmed the previous results of another laboratory that showed expression in CNS neurons. Unlike CB_1_ cannabinoid receptors, which are expressed in almost any neuron, the CB_2_ is only expressed in certain types of neurons in specific regions of the CNS [58,59,60,61,62,63,64,65,66,67]. When we discovered the differential expression of two olfactory receptors in activated microglial cells in response to neuroprotective adenosine receptor ligands [68,69], we were interested in ascertaining the following: (1) whether olfactory receptors are involved in the pathophysiology of neurological diseases associated with neuroinflammation, (2) whether olfactory receptors were expressed in neurons outside the olfactory system, and (3) whether olfactory receptors were expressed in peripheral organs and tissues.

### 5.1. Ectopic Expression of Olfactory Receptors in Peripheral Tissues and Organs

Accumulated evidence suggests that olfactory receptors may not be solely related to odors. About three decades ago, the laboratory of Parmentier cloned a gene with high expression in the dog testis. The gene has 82% identity with OLF15, a gene cloned from the rat olfactory mucosa, and its product is known as “*olfactory receptor-like protein DTMT*” [70]. Odor receptor-like proteins were also identified decades ago in spermatids from rat testes; they were called spermatid chemoreceptors [71]. Subsequent studies in the mouse showed that an olfactory receptor plays a relevant role in sperm chemotaxis and participates in the regulation of sperm motility [72]. Sperm capacitation is also regulated by an olfactory receptor, OR2C1 [73]. Up to 91 transcripts corresponding to human OR genes have been identified in human sperm. Very notable was the concomitant finding of “*putative OR transcripts in an antisense orientation, indicating a different function, rather than coding for a functional Olfactory receptor protein*” [74].

Comprehensive details of the non-chemosensory organs and tissues in which olfactory receptors have been identified are beyond the scope of the present review. All tissues likely express some of the hundreds of proteins known as olfactory receptors. From the kidney to the intestine and muscle, olfactory receptor expression has been described (see [75] for review). OR gene transcripts are found in almost all transcriptomics studies. Deep sequencing data available for the human testes and 16 other tissues from the Illumina Body map 2.0 project showed that some OR genes were expressed in all the tissues analyzed, and that the OR51E1 and OR2W3 genes were also widely expressed. A total of 111 transcripts for different olfactory receptors were found “ectopically”. Only one OR gene, OR4N4, was exclusively expressed in the human testes [76].

The exact function of ectopic olfactory receptors is often unknown. But when partially deciphered, it correlates with important functions such as tissue repair; in fact, the mouse MOR23 receptor participates in regeneration and the regulation of cell adhesion and migration in muscle [77]. Of those identified by bioinformatic analysis of the transcriptome of human eccrine sweat glands, researchers confirmed the appearance of OR51A7 and OR51E2 by in situ hybridization and immunohistochemistry. The function of these two olfactory receptors appears to be the regulation of sweat production, as deduced from the results of receptor activation using β-ionone [78]. Olfactory receptors expressed in the kidney can fulfill different functions related to the control of blood vessel tone. One study reported that in mice, Olfr78 in the kidney responds to compounds produced by the microbiota and regulates the renin–angiotensin system which, in the periphery, is primarily engaged in blood pressure control [79]. Olfr558 is expressed in enterochromaffin to detect metabolites produced by the microbiota; the function of the receptor appears to be to regulate the production of serotonin by these cells and, in doing so, provide information to the neurons of the afferent nerve fibers [80]. Human skin cells express OR2AT4, which appears to be involved in wound healing and re-epithelialization [81].

The role of the dozens of receptors detected in the cells of the human reproductive system must correspond to very important functions, which are those that underlie the most important survival event of mammals: reproduction. It can be hypothesized that they may contribute to sperm chemotaxis. Interestingly, it is suggested that steroids (sex hormones are steroids) can interact with olfactory receptors and eventually activate them. The G protein-coupled receptor database (https://gpcrdb.org/) lists, accessed on 30 March 2024, 73 possible activators of OR51E2, 2 of them being epitestosterone and estriol. This information creates the following two interesting possibilities: (1) estrogen can activate G protein-coupled receptors on the cell surface and (2) these receptors may belong to the olfactory family. Whereas estriol is endogenously produced, humans are exposed to epitestosterone, a natural compound found in human urine and is supposed to regulate events driven by androgens such as body hair distribution and control of prostate growth [82].

### 5.2. Olfactory Receptors in Neurons and Glia of the CNS

Olfactory receptors expressed in olfactory sensory neurons have been implicated in more functions than just the identification of odorants. These functions encompass axonal guidance and plasticity related to the previous exposure to odorants [83,84,85]. The presence of olfactory receptors in the autonomic nervous system ganglia supports the notion that they serve functions beyond odorant detection, including the response to molecules endogenously produced [86,87].

Relevant studies have brought to light that olfactory receptors exhibit extensive distribution within the human central nervous system (CNS). This distribution encompasses the cerebral cortex, thalamus, specific brainstem nuclei, and Purkinje cells, particularly for certain receptors (OR2H, OR2A4, and OR6K3) [88]. Olfactory receptors have been detected in the substantia nigra and ventral tegmental area of mesencephalic dopaminergic neurons, areas that play an important role in Parkinson’s disease and schizophrenia, respectively; these receptors are functional in responding to odorant-like compounds [14].

Our interest in olfactory receptors began with the identification of two members of two different families in mouse microglia. Transcriptomics analysis showed differential expression of the genes of the two olfactory receptors in the activated microglia [68,69]. The function of olfactory receptors in the glia is not known, but one possibility is to act as regulators of the function of other G protein-coupled receptors. By receptor–receptor interactions, G protein-coupled receptors can form heteromers with a different functionality than that of the receptors in a non-heteromeric context. The human ortholog of one of the olfactory receptors detected in microglia may interact with the human adenosine A_2A_ receptor [89], which is expressed in the microglia and is a target for neuroprotection [90,91,92,93]. It should be noted that A_2A_ receptor expression is upregulated in the microglia surrounding the aberrant structures found in the brain of Alzheimer’s disease patients [94].

### 5.3. Ectopic Olfactory Receptors in Alzheimer’s and Parkinson’s Diseases

Pioneering studies involving PCR and immunohistochemistry showed that altered olfactory receptor expression in the frontal cortex appears at relatively early stages of the neurodegenerative processes in Parkinson’s disease [15,88,95].

The prevailing focus in the literature concerning smell disorders in neurodegenerative diseases, specifically Parkinson’s disease, centers on the presence of altered molecules in the olfactory tract and the modification of complex functional connectivity in the primary and secondary central olfactory areas. The issue became more complex when differential expression of olfactory receptors was detected in non-chemosensory areas of the CNS of patients [88].

Gene expression analysis and subsequent validation by quantitative PCR in human samples has unveiled a cluster of olfactory receptor genes that are differentially expressed in the frontal cortex of Parkinson’s disease patients. Such alterations manifest relatively early in the disease, emerging at premotor stages, suggesting that it may manifest in the early phases of the pathology. The cause is not drug therapy, because in the aforementioned study, the subjects in premotor stages had not received medication [88]. Intriguingly, the expression of the transcript for the OR4F4 receptor increases exclusively in women diagnosed with Parkinson’s disease [13,88]. Concerning the substantia nigra, Parkinson’s disease is associated with the downregulation of certain olfactory receptors [88]. This intricate interplay of gene expression alterations sheds light on the multifaceted aspects of disease pathogenesis and leads to placing olfactory receptors on the stage of neurodegenerative diseases.

The expression of the transcripts of the genes of a pair of olfactory receptors, OR4F4 and OR11H1, of the eight investigated, was altered in the entorhinal cortex of patients with Alzheimer’s disease in different Braak and Braak stages. In the cortex of the APP/PS1 transgenic mice model of the disease, there was a progressive increase in Olfr110 mRNA with aging (from 3 months onwards) [96]. In the mice CNS, Olfr110/111 and Olfr544 were discovered in the cortical and hippocampal neurons, astrocytes, microglia, oligodendrocytes, and endothelial cells. The expression of the genes for these receptors is deregulated in the 5xFAD model of Alzheimer’s disease, which is a transgenic mouse carrying five mutations linked to the familial disease. At the protein level, Olfr110/111 and Olfr544 were found associated with nearby amyloid plaques. Interestingly, a compound secreted by Streptococcus pneumoniae, 2-pentylfuran, activates mouse microglia via Olfr110/111 [97]. Taken together, olfactory receptors may contribute to the regulation of microglial activation and, if confirmed, could have potential in the context of anti-neuroinflammatory drug development [98].

A transcriptomics study in peripheral blood polymorphonuclear mononuclear cells showed that the expression of two olfactory receptors, OR11H1 and OR4M1, correlated with the severity of traumatic brain injury. The authors continued to address the expression of OR genes in the brain and found ectopic expression in the entorhinal hippocampus and other areas relevant to memory processing and consolidation. Furthermore, phosphorylation of the tau residues that appear hyperphosphorylated in Alzheimer’s disease was reduced after the activation of OR4M1 by acetophenone [99].

Olfactory receptors in non-sensory areas of the CNS probably act as chemoreceptors and have endogenous ligands that need to be identified. Dysregulation of ligand production and expression and the function of ectopic CNS olfactory receptors may have implications for the pathophysiology of neurodegenerative diseases. Future research may provide insights into the pathophysiology of the disease while leading to new therapeutic avenues.

## Data Availability

Data may be provided by the corresponding author upon reasonable request.
